# Aligning Funding and Need for Family Planning: A Diagnostic Methodology

**DOI:** 10.1111/sifp.12034

**Published:** 2017-10-17

**Authors:** Victoria Y. Fan, Sunja Kim, Seemoon Choi, Karen A. Grépin

## Abstract

With limited international resources for family planning, donors must decide how to allocate their funds to different countries. How can a donor for family planning decide whether countries are adequately prioritized for funding? This article proposes an ordinal ranking framework to identify under‐prioritized countries by rank‐ordering countries by their need for family planning and separately rank‐ordering them by their development assistance for family planning. Countries for which the rank of the need for family planning is lower than the rank of its funding are deemed under‐prioritized. We implement this diagnostic methodology to identify under‐prioritized countries that have a higher need but lower development assistance for family planning. This approach indicates whether a country is receiving less compared to other countries with similar levels of need.

The societal benefits of family planning services include the direct effects of reduced fertility and improved health of women and children, the indirect effects of increased women's earnings and employment, and the economic and demographic effects of reduced fertility (Canning and Schultz 2012). Many countries have yet to reap these benefits by expanding access to family planning, which will require financing from domestic and international sources. International donors have highlighted the importance of family planning, as reflected by the Family Planning 2020 initiative (hereafter FP2020), which aims to accelerate the pace at which women gain access to high‐quality, modern family planning and reproductive health services (Brown et al. 2014; FP2020 and United Nations Foundation 2014).

With limited budgets for family planning, donors and national governments must choose how to spend their funds. In particular, donors must decide how to allocate their funds for a portfolio of countries in order to reach certain pre‐defined objectives. One study has examined the determinants of assistance for population and reproductive health with a particular focus on 21 bilateral donors and individual categories of funding (van Dalen and Reuser 2006). Another study has examined reproductive health funding by 31 donors to 75 countries chosen for the Countdown to 2015 for Maternal, Newborn, and Child Survival (Arregoces et al. 2015). The study analyzed the inter‐country variation in assistance for maternal and newborn health per woman of reproductive age, or in assistance for child health per child. It assessed the overall association of selected measures of need (under‐5 child mortality rate, maternal mortality ratio, HIV prevalence, and female life expectancy at birth) with reproductive health assistance and found a strong correlation between need and funding. These two studies, however, did not directly ask whether countries that need family planning funding are receiving adequate assistance.

The objective of the present study is to assess patterns in the distribution of funding vis‐à‐vis a given indicator of need and to determine which countries may not be receiving adequate assistance according to the chosen indicator. This diagnostic methodology is applied to the countries receiving development assistance for family planning. The methodology is not definitive, because donors and countries can have differing priorities for development assistance for health (Grépin et al. 2017). Our objective is to identify those countries that are receiving lower‐priority assistance given a chosen set of indicators of need, regardless of country and donor objective. Although this methodology uses conventional indicators of need for family planning, we do not claim that those indicators ought to be shared by all parties or that there is a global goal that ought to be adopted by all countries.

## DIAGNOSTIC METHODOLOGY

Allocation methodologies can be categorized as one of three types: a production function, a budget model, and a spending function (Fan et al. 2014). Each of these three types has strengths and weaknesses, and there is disagreement on the numerical precision and the assumptions underlying these models (Collier and Dollar 2002; Leo 2010). For example, the production function assumes that there is a universal function to produce an outcome (e.g., contraceptive use), whereas the budget function requires data on the costs of programs. Moreover, these frameworks assign some level of optimal allocation to which de facto levels of allocations are compared. Researchers using these approaches often assume that the chosen objective is universal, when in practice donors and countries have their own goals and objectives and their preferred indicators of need (Ottersen et al. 2017; Grépin et al. 2017).

Past work on allocation methodologies can be characterized as taking a cardinal approach to identifying worse‐off countries, which lends itself to making definitive prescriptions about whether countries are getting enough funding and, if not, the extent of their shortfall. In contrast, we use an ordinal approach by rank‐ordering funding vis‐à‐vis need (Sen 1976). An ordinal approach recognizes that there may be agreement on which countries are worse off, but this approach does not require agreement on the numerical values of those differences. This approach does not take a stance about the extent to which a country might be inadequately supported, but simply points to whether a country is getting less compared to other countries with similar levels of need.

*Rank‐order countries, separately by need and by funding*. We first rank‐order countries in terms of the country's need for family planning, with higher ranks indicating greater need. We denote this as the country's *need rank*. The chosen indicators are defined in terms of either achievement or a gap. A country's need rank, for example, would be higher both for countries with a lower prevalence of contraceptive use and for those with a higher population growth rate. Next, we rank‐order countries in terms of the country's received assistance for family planning, with larger ranks indicating higher levels of funding. We denote this as the country's *funding rank*, and we assume that countries with higher need rank should therefore have a higher funding rank.

*Calculate the rank differences for each indicator*. For each country, we subtract the country's need rank from its funding rank, denoted as the country's *rank difference*. A country whose need rank is higher than its funding rank has by definition a negative rank difference. These countries are therefore potentially under‐funded relative to their need. Conversely, a positive rank difference indicates that the country has lower need compared to its funding. We implement this framework of country rank differences for a given indicator of need to identify countries that are potentially under‐prioritized. We label this outcome as only potentially under‐prioritized because (1) each difference considers only a given measure of need, (2) donors and countries have different preferences for what constitutes need for family planning, and (3) donors and countries may have multiple indicators of need as objectives for their family planning assistance. This rank difference does not simultaneously rank multiple indicators.

*Calculate a summary score*. We then generate a summary score by combining these rank differences for multiple indicators. For each indicator of need, we generate a binary indicator in which a country that ranks in the bottom 10 percent of the rank difference is assigned 1 and 0 otherwise. In other words, countries assigned the value of 1 have larger *negative* rank differences compared to those with 0. These binary measures of need effectively list the worst‐off countries. We then sum these multiple binary indicators to generate a summary score by counting each indicator for which the country is in the bottom 10 percent. The summary score is simply the number of indicators for which the country is in the bottom 10 percent. The methodology depends on the threshold that one chooses—in our case, the bottom 10 percent. The lower the threshold, the greater the concern about the country's larger rank difference. As a robustness check, we used a higher threshold of 25 percent, which includes countries with smaller rank differences.

*Choose indicators of need*. Implementing this methodology requires the choice of indicators of need. The results of our methodology should be interpreted indicatively rather than definitively, since not all donors will agree with our choice of indicators. Those donors or countries that agree with our indicators as their objectives would be more inclined to agree with the findings of which countries have higher need. Our methodology is replicable in that donors with a focus on a given indicator can apply the framework.

We have chosen a broad set of family planning indicators and individual measures of need that have been prioritized by various donor agencies, including the three primary indicators of the US Agency for International Development (the predominant funder of family planning), a core indicator of the Sustainable Development Goals, and other indicators that have been articulated as broad objectives of family planning assistance (Silverman and Glassman 2016).

For this study we selected measures of contraceptive use, population growth and structure, maternal mortality, and gender inequality. Core indicators were reviewed by 26 stakeholders including the three largest donors (USAID, UK DfID, and the Bill and Melinda Gates Foundation), as well as other partners, implementers, and researchers (Silverman and Glassman 2016), and additional indicators were chosen through peer review. We describe the specific rationale for choosing each indicator.

## DATA

### Data on Need for Family Planning

Our four main indicators of need for family planning are the (1) modern contraceptive prevalence rate, (2) annual population growth rate, (3) maternal mortality ratio, and (4) Gender Inequality Index. The first three indicators were used by USAID as part of its allocation process (“USAID Resource Guide for Family Planning,” n.d.). We included the Gender Inequality Index in response to USAID's recommendation to promote gender equality and integrate women's empowerment approaches into family planning (ibid.). Given the importance of the Sustainable Development Goals, we also included the percent of the population under age 15, which is part of the denominator of the dependency ratio and an indicator of the demographic dividend. As a complement to the population growth rate, we included the total fertility rate, which is independent of age structure. And as a complement to contraceptive prevalence, we included percent of demand for modern contraceptives satisfied and unmet need for modern contraception.

*Contraceptive use and demand*. The United Nations Population Division (2015) defines the modern contraceptive prevalence rate (mCPR) as the number of married/in‐union women aged 15–49 years using a modern form of contraception (henceforth female contraceptive users) divided by all married and/or in‐union women aged 15–49. Percent of demand for modern contraceptive use satisfied is defined as the percent of women of aged 15−49 who are sexually active and have their need for family planning satisfied with modern methods (ibid.). Unmet need for modern contraceptives is defined as the percent of married or in‐union women aged 15–49 who want to stop or delay childbearing but are not using modern contraception (Bradley et al. 2012). The data for mCPR and percent of demand for modern contraception satisfied are calculated by and obtained from the United Nations Population Division (Alkema et al. 2013; United Nations Population Division 2016).

*Population growth and structure*. The annual population growth rate is the exponential rate of growth of midyear population expressed as a percentage (United Nations Population Division 2015). The percent of the population under age 15 is the number of people younger than 15 divided by the total population in the country (World Bank 2016). The total fertility rate is the number of children who would be born per woman if she were to pass through the childbearing years bearing children according to current age‐specific fertility rates. The data for these indicators were obtained from the World Bank open database (ibid.).

*Maternal mortality ratio*. The maternal mortality ratio (MMR) is the number of maternal deaths from pregnancy‐related causes per 100,000 live births among women aged 15–49 (Alkema et al. 2016). The data for this indicator were produced by the United Nations Maternal Mortality Estimation Inter‐Agency Group and downloaded from the World Bank database (Alkema et al. 2016; World Bank 2016).

*Gender Inequality Index*. The Gender Inequality Index (GII) measures gender inequality in reproductive health, empowerment, and economic status. This index, generated through the United Nations Development Programme's Human Development Reports, is indicative of systematic disadvantages of women in certain countries and areas (United Nations Development Programme 2015). For 17 countries for which GII was not available for 2014, we computed the index using the tool provided online for each of the countries (United Nations Development Programme 2016).

### Data on Development Assistance for Family Planning

The Organization for Economic Co‐operation and Development (OECD) defines official development assistance (ODA) as financial or in‐kind contributions provided by official governmental agencies (bilateral or multilateral) to developing countries for improving economic development and welfare. Apart from ODA, the international flow of funds to developing countries includes other official flows (OOF) and private flows (such as those from private foundations). Here we refer to development assistance for health (DAH) as including ODA, OOF, and private grants for all health areas including population and reproductive health and family planning. We rely on two databases to examine development assistance for family planning between 2004 and 2014: the OECD Creditor Reporting System (OECD‐CRS) (OECD 2015) and the International Aid Transparency Initiative (IATI) (International Aid Transparency Initiative, n.d.).

*OECD‐CRS*. The OECD‐CRS is one of the most widely used datasets on foreign aid flows. The OECD‐CRS for 2004–14 is publicly available and the most recent update was downloaded in January 2016. The database provides information on recipient countries, regions, type of aid, purpose and sector classification, dates, project title, and descriptions. We used a purpose/sector code to restrict project data to development assistance for family planning (5‐digit purpose code 13030). All dollar amounts in OECD‐CRS are reported in US dollars. In the OECD‐CRS, donors report their bilateral ODA, multilateral ODA, earmarked ODA channeled through a multilateral organization (referred to as bi/multi), OOF, and private flows. To avoid double counting, the OECD‐CRS does not include a donor's core contributions to multilateral agencies. Instead, a multilateral organization's (un‐earmarked) flows to a recipient country are reported as multilateral ODA. ODA earmarked by a bilateral organization through a multilateral channel is classified under bilateral ODA.

*IATI*. The IATI database is similar to the OECD‐CRS in the variables used, but, unlike the OECD‐CRS, IATI has a time‐series from 2004 of development assistance for health by the Bill and Melinda Gates Foundation (BMGF), a private foundation with significant contributions to global health and in particular family planning. We use IATI data for BMGF, adjusting currency and appending it to the OECD‐CRS data. Data from IATI were preferred over data from publicly available BMGF reports because of the consistency of sector/purpose coding with that of the OECD‐CRS as well as the structure of the data and the number of variables.

The OECD‐CRS (ODA) plus IATI‐BMGF (private flows) database was the main database analyzed for this article (henceforth referred to as the OECD‐CRS+IATI‐BMGF database). When we refer to DAH, we are referring to this specific database, which excludes all OOF and other private flows (i.e. except BMGF). All disbursement figures are shown in constant 2013 US dollars.

## RESULTS

### Descriptive Statistics of Family Planning Assistance, 2004–14

**Figure 1:**
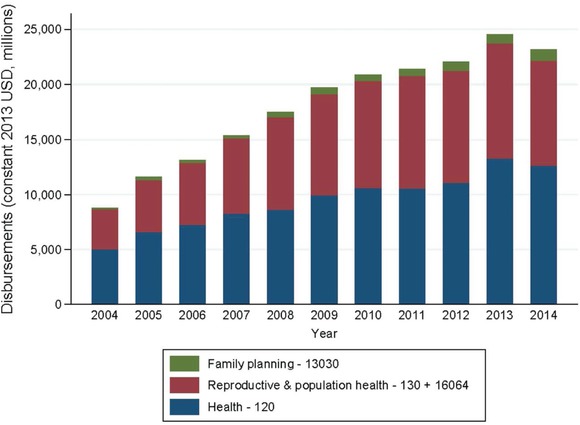
Disbursement for development assistance for health by CRS sector, 2004–14 NOTES: Authors' calculations. Reproductive and population health (130 + 16064) here excludes family planning. The number 13030 refers to the CRS purpose code for family planning funds; 130 refers to the Development Assistance Committee sector block (DAC5 code) for population policies/programs and reproductive health; 16064 refers to the CRS purpose code for social mitigation of HIV/AIDS; 120 refers to the DAC5 code for health.

Family planning represented a small fraction of total DAH, but that fraction has grown faster than other areas of DAH. Family planning accounted for 3.0 percent of total DAH over 2004–14. DAH increased in absolute levels over time from $8.8 billion in 2004 to $23.2 billion in 2014, a 3.8‐fold increase. In contrast, assistance for family planning increased from $134 million to $1.0 billion, a 13.3‐fold increase. Assistance for reproductive health (130+16064, excluding family planning) increased from US$3.7 billion in 2004 to $9.6 billion in 2014, a 3.8‐fold increase (Figure 1).

**Figure 2:**
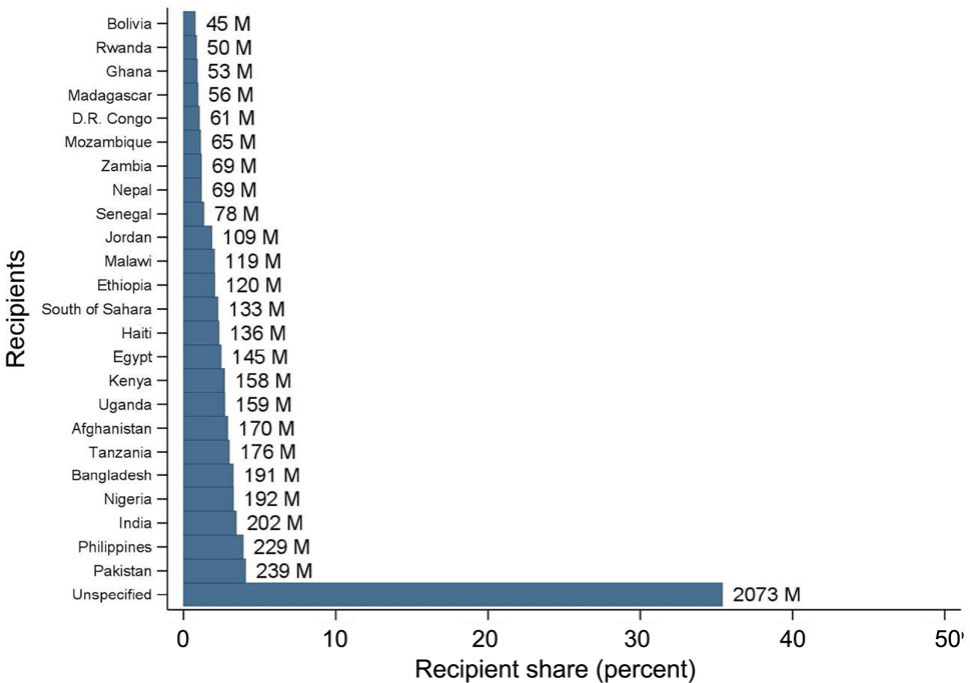
Recipient share in global family planning disbursements, 2004–14: Top 25 recipients NOTE: Authors' calculation. M = disbursement in millions of constant 2013 US dollars.

Total DAH for family planning over 2004–14 was $5.86 billion. Disaggregated by donor, the United States was the largest at $4,113 million (70.2 percent), followed by the United Kingdom at $600 million (10.2 percent) and BMGF at $484 million (8.3 percent). These three donors represent 88.7 percent of development assistance for family planning, indicating a highly concentrated donor industry. Given that the US and UK together account for 80 percent of all family planning assistance, we restrict our analysis to the 76 countries that receive assistance from these two donors or are otherwise prioritized by FP2020. The major recipients of family planning assistance (in millions) were Pakistan ($239), Philippines ($229), India ($202), Nigeria ($192), and Bangladesh ($191), representing 29 percent of all country‐specified family planning disbursements over 2004–14 (Figure 2). The top 20 recipients received 76 percent of family planning disbursements.

### Implementation of Diagnostic Methodology

We first examine correlations of disbursements per capita with each indicator of need. We then convert the underlying cardinal data to ordinal data to calculate the country rank differences for each indicator of need. We present these rank differences for each of the four indicators of need. We then present the summary scores that count the number of indicators for which the country is listed in the bottom 10 percent of the rank difference.

**Figure 3:**
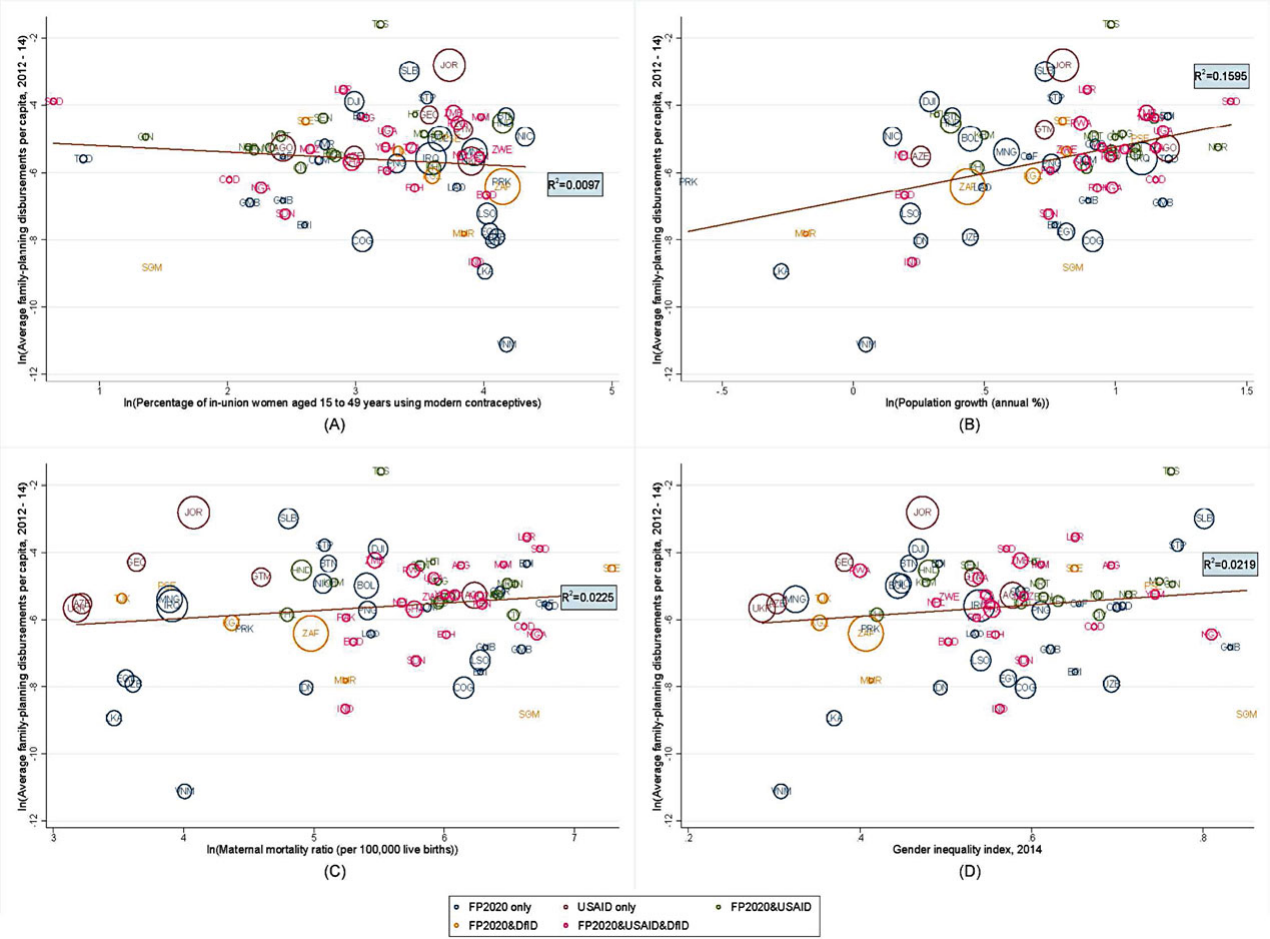
Average family planning disbursements per capita plotted against indicator of need, 2012–14 NOTE: Authors' calculations. The color‐coding represents the prioritization of these countries by FP2020, USAID, and/or DfID. The bubble size is weighted based on relative government health expenditure per capita (World Bank 2016). The countries are labeled with the ISO Alpha‐3 codes.

Figure 3 presents the crude correlation of a country's average family planning disbursements per capita received over 2012–14 against each indicator of need. The figure plots the country's level of family planning assistance disbursements per capita on the y‐axis and the value of the country's individual indicator of need on the x‐axis. Panel A shows the slightly negative crude correlation between mCPR and disbursements per capita. The figure indicates that as the mCPR rises (with higher coverage), a country might be expected to receive a lower disbursement. Panels B, C, and D indicate a positive correlation between family planning disbursements per capita and each indicator of need. Thus, countries with a higher population growth rate, higher MMRs, and higher GII scores tend to receive more family planning funding per capita.

**Figure 4:**
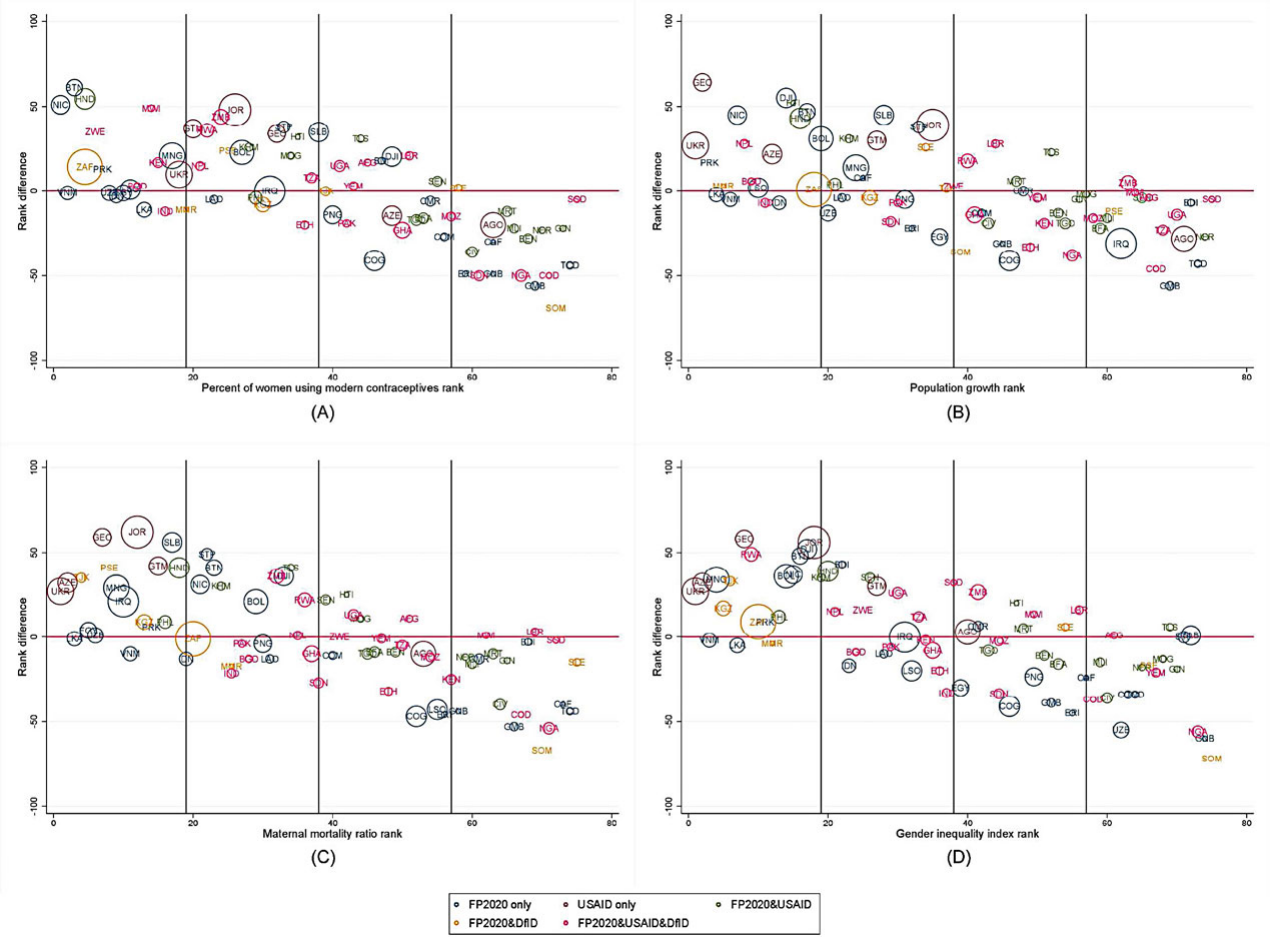
Difference in rank of family planning disbursements per capita and rank of indicator of need NOTE: Authors' calculations. The color‐coding represents the prioritization of these countries by FP2020, USAID, and/or DfID. The bubble size is weighted based on relative public/government health expenditure per capita (World Bank 2016). The countries are labeled with the ISO Alpha‐3 codes.

To implement our diagnostic methodology, we transform indicators of need and indicators of funding into ranks and calculate the rank difference. Figure 4 shows each country's rank difference (funding rank minus need rank) on the y‐axis. The more negative the rank difference, the larger the need rank compared to the funding rank. The figure also suggests that countries with larger per capita government health financing (larger bubbles) also have smaller rank differences. A country may have a high rank for need, but a low rank for funding, resulting in a large rank difference even if the level of need is relatively low. Somalia (SOM) repeatedly appears under‐prioritized with the most negative rank difference for mCPR (Panel A), MMR (Panel C), and GII (Panel D). Panel B shows that Gambia (GMB) appears under‐prioritized given its population growth rate, with the most negative rank difference.

Next, we calculate two summary scores. One score uses mCPR, population growth rate, and MMR (Table 1, Panel A), the other includes GII with the other three indicators (Table 1, Panel B). The panels show only those countries that had at least one indicator in the bottom 10 percent of the rank difference. For example in Panel A, D.R. Congo ranks in the bottom 10 percent of the largest negative rank difference for all three indicators and hence has a summary score of 3. Table 1 contains countries that could be seen as under‐prioritized in terms of family planning disbursements per capita. Five countries appeared in the bottom 10 percent of the rank difference for all four indicators: D.R. Congo, Gambia, Guinea‐Bissau, Nigeria, and Somalia.

**Table 1:** Country summary scores aggregating multiple indicators of need

Country	Score
**Panel A: Aggregating 3 indicators** a	
D.R. Congo	3
Gambia	3
Guinea‐Bissau	3
Nigeria	3
Somalia	3
Chad	3
Congo	2
Eritrea	2
Ethiopia	1
Iraq	1
Sudan	1
**Panel B: Aggregating 4 indicators** b	
D.R. Congo	4
Gambia	4
Guinea‐Bissau	4
Nigeria	4
Somalia	4
Congo	3
Eritrea	3
Chad	3
Ethiopia	1
Iraq	1
Sudan	1
Uzbekistan	1

Note: Authors’ calculations. Panel A aggregates rank differences for mCPR, population growth rate, and MMR. Panel B adds the GII.

a Maximum score = 3;

b Maximum score = 4.

There are 11 countries in Panel A and 12 in Panel B, which includes Uzbekistan due to the rank difference for its GII. All of the countries are in sub‐Saharan Africa except Iraq and Uzbekistan, and all except Uzbekistan are FP2020 focus countries.

## DISCUSSION

We have identified recipient countries that appeared to be under‐allocated according to multiple indicators of need for family planning. One advantage of this study is that it examines funding sources from multiple donors. While donors may prioritize funds according to their own preferences and objectives, the collective allocation by donors may neglect certain countries. Thus, this analysis identifies under‐allocated countries using indicators that are valued by many in the family planning community (though we recognize that such indicators are not necessarily universal and exhaustive).

Determining why these countries have relatively low disbursements per capita compared to countries with similar levels of need merits further research. While fragile countries share a number of characteristics explaining their relatively low investment, other fragile states, such as Afghanistan, Haiti, Liberia, and Madagascar, were not identified as under‐prioritized by this diagnostic tool. Thus, our methodology identifies those countries that are getting less relative to an indicator of need. A main policy recommendation of this study is that donors re‐examine their allocation procedures, consider their overall portfolio, and decide whether re‐allocations and/or better coordination among multiple donors (e.g., through multilateral institutions) is merited. This diagnostic tool can be used to highlight inequalities in allocation.

This diagnostic methodology should not be the only means of allocating funding, as it relies on only four indicators of need. Of course, some countries confront challenges in implementation, hence re‐allocating a greater share of funding to these countries may not yield greater value for money. Other relevant factors in terms of value for money include domestic health financing, financing for family planning in particular, per capita income, government effectiveness, weak governance as designated by the label of fragile or conflict state, or other factors that might affect how much funding a country receives for development assistance for family planning. It remains to be seen whether investing in fragile states, in either the short or long run, is good value for money.

As a robustness check, given that the chosen indicators do not universally reflect all donors, we have carried out additional analyses of four other major family planning indicators (see Supplementary materials,^1^ Tables S1 and S2 and Figures S1 and S2): (1) unmet need for modern contraceptives, (2) demand for modern contraceptives satisfied, (3) percent of population under 15 years, and (4) total fertility rate (“Family Planning and Reproductive Health Indicators Database,” n.d.). When Table S1 is compared to the results from Panel B of Table 1, which presents the mCPR, population growth rate, MMR, and GII ranks, only D.R. Congo and Somalia have similar summary scores. However, since the indicators of interest are different, the recipient countries that are considered under‐allocated for family planning differ as well.

We carried out other robustness checks. First, we used a threshold of the bottom 25 percent rather than the bottom 10 percent in calculating a summary score (see Table S3). Our use of a more generous threshold identified more countries for prioritization. Second, we examined indicators of need using ranks of absolute amounts (number of contraceptive users, population growth, and number of maternal deaths) against ranks of total disbursement amounts (not per capita) (Tables S4 and S5). We found greater correlation between absolute levels of need and total family planning disbursements than between proportional need and disbursements per capita, as shown by the fact that no country was in the bottom 10 percent of the rank difference for all three indicators (Table S4).

There are significant limitations to using data on donor allocations. Classifying funds as family planning versus reproductive health is not trivial. As such, we carried out a robustness check by re‐calculating the summary score using the broader category of reproductive health and separately the category of reproductive health and health systems strengthening (Figures S3–S6 and Tables S6 and S7). Nevertheless, these results are similar to our main findings using family planning disbursements per capita, with a longer list of countries (17 and 18 countries for reproductive health, and reproductive health with health systems strengthening, respectively, compared to 12 for family planning).

A second limitation related to the data was the high proportion of funds that were assigned a value of unallocated/unspecified, a challenge common to data for all sectors for development assistance for health. This broad recipient category makes it difficult to determine which countries and organizations benefited from these funds. These funds were therefore not included in our analysis. We made no assumptions about country distributions for this unallocated amount, and more work is needed to understand what these costs entail.

There are other limitations to this study. We did not disaggregate the indicators of need by potential covariates of interest, namely, education level, wealth quintile, age, and urban‐rural residence. Doing so could help us to identify countries or populations that might see better return on investment. This study also assumes that countries with higher need ought to have higher funding. In practice, however, this might not be an ideal strategy. For example, a country with a low mCPR might be expected to receive a higher disbursement per capita. As the country's mCPR rises, the amount per capita needed could be lower if we assume certain economies of scale as costs of delivery decrease. On the other hand, as a country's mCPR rises, it might be more costly to reach those women who do not yet use modern contraceptives, so the amount per capita might need to remain high at least for those populations. Hence, it is debatable whether a country with a lower mCPR (and therefore a higher rank in mCPR) should have higher family planning disbursements (and therefore a higher funding rank). This uncertainty about the underlying cost function of delivering family planning services is a significant limitation of the study (but also for studies using the budget function). Our diagnostic methodology should not be erroneously used on its own to conclude that donors ought to increase their funding levels to under‐prioritized countries. Instead, we recommend that donors identify the reasons for under‐prioritization, seek to address challenges of reaching those populations, and further quantify the cost functions of the delivery of family planning services. At the same time, we urge donors not simply to dismiss those countries that are deemed to be fragile, as we identified some fragile states that were not under‐prioritized.

Donors who choose to apply this diagnostic methodology to their own allocation portfolio should select the indicators that reflect their objectives, which vary by donor and country. The methodology's ranking approach is simple, transparent, and replicable. While our approach is neither definitive nor sufficient on its own, it can be used in conjunction with other methodologies. Despite the numerous caveats of the methodology and limitations to data, this diagnostic methodology can help to address inequalities in funding, not only in family planning but in other areas as well.

## Supporting information

Robustness check using different indicators of need and funding

## Footnotes

1 Supplementary materials are available at the supporting information tab at wileyonlinelibrary.com/journal/sfp.
