# Supplementary material for: Aligning Funding and Need for Family Planning: A Diagnostic Methodology
Source: Stud Fam Plann. 2017 Oct 17;48(4):309–22. doi: 10.1111/sifp.12034 (PMC5725659; doi:10.1111/sifp.12034)
Supplement: Supplementary file 1 — Robustness check using different indicators of need and funding [file SIFP-48-309-s001.docx]

# **SUPPLEMENTARY MATERIALS**

## Robustness check using different indicators of need and funding

### Table S1. Country summary scores (maximum = 4) aggregating unmet need for modern contraceptives, demand for modern contraceptives satisfied, population under 15 years, and total fertility rate using 10% threshold

| **Country** | **Score** |
| --- | --- |
| D.R. Congo | 4 |
| Congo | 4 |
| Somalia | 4 |
| Eritrea | 3 |
| Gambia | 3 |
| Nigeria | 3 |
| Guinea-Bissau | 2 |
| Chad | 2 |
| Azerbaijan | 1 |
| Cote d'Ivoire | 1 |
| Comoros | 1 |
| Ethiopia | 1 |
| Ghana | 1 |
| Philippines | 1 |
| Sudan | 1 |

### Table S2. Country summary scores (maximum = 4) aggregating unmet need for modern contraceptives, demand for modern contraceptives satisfied, population under 15 years, and total fertility rate using 25% threshold

| **Country** | **Score** |
| --- | --- |

|  |  |
| --- | --- |
| D.R. Congo | 4 |
| Congo | 4 |
| Eritrea | 4 |
| Gambia | 4 |
| Sudan | 4 |
| Somalia | 4 |
| Angola | 3 |
| Cote d'Ivoire | 3 |
| Comoros | 3 |
| Guinea-Bissau | 3 |
| Mali | 3 |
| Nigeria | 3 |
| Chad | 3 |
| Azerbaijan | 2 |
| Benin | 2 |
| Burkina Faso | 2 |
| Central African Republic | 2 |
| Ethiopia | 2 |
| Ghana | 2 |
| Mozambique | 2 |
| Niger | 2 |
| Togo | 2 |
| Tanzania | 2 |
| Bolivia | 1 |
| Egypt | 1 |
| Guinea | 1 |
| Iraq | 1 |
| Sri Lanka | 1 |
| Lesotho | 1 |
| Pakistan | 1 |
| Philippines | 1 |
| Papua New Guinea | 1 |
| Uganda | 1 |
| Yemen | 1 |

### Figure S1**. Average family planning disbursements per capita vs. indicator of need, 2012-14**


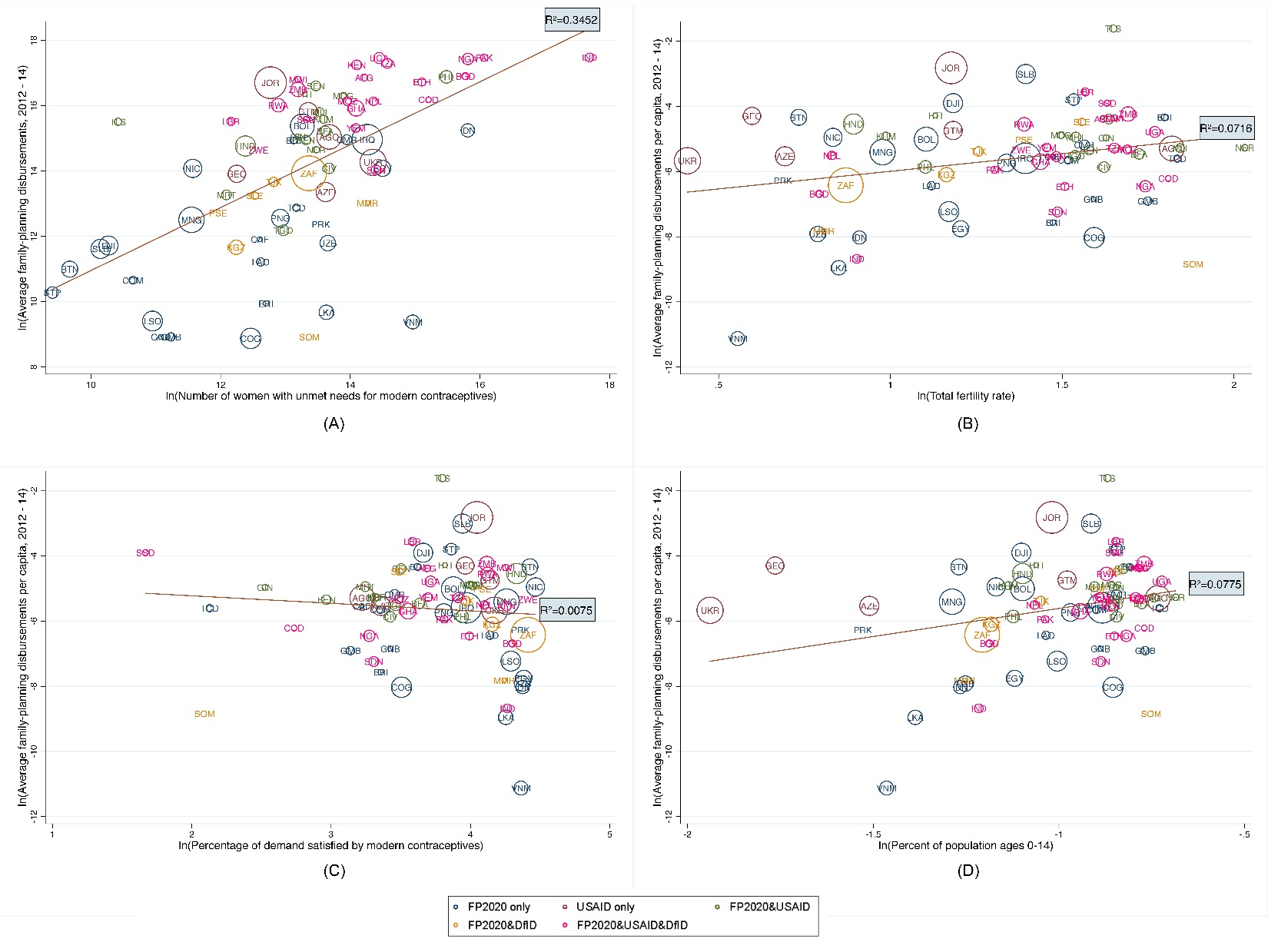


### Figure S2. Difference in rank of family planning disbursements per capita and rank of indicator of need

**
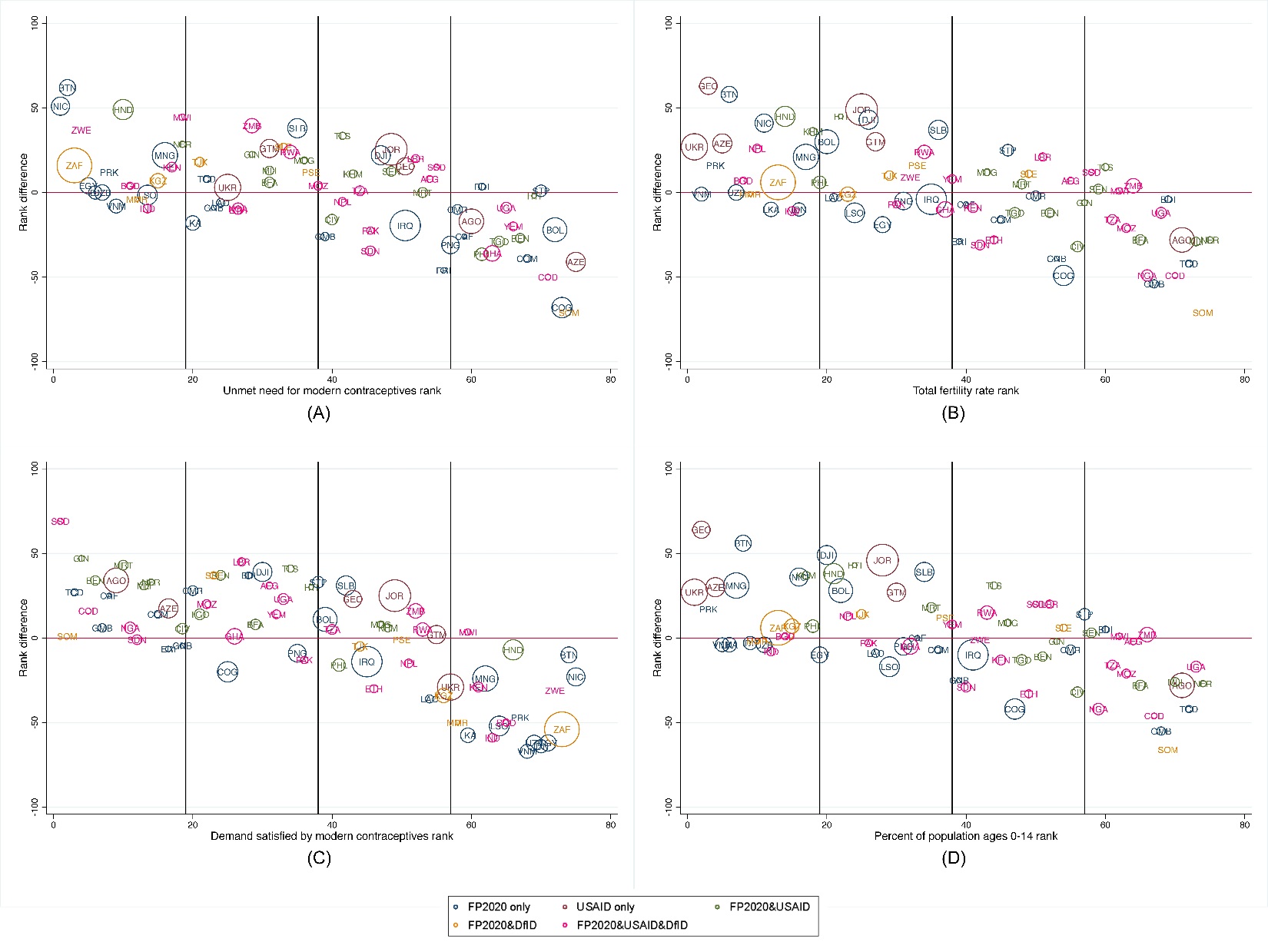
**

## Robustness check using 25% threshold

### Table S3. Country summary scores aggregating multiple indicators of need using 25% threshold

| **Country** | **Score** |
| --- | --- |
| *Panel A: Aggregating 3 indicators of need^a^* | |
| Cote d'Ivoire | 3 |
| D.R. Congo | 3 |
| Congo | 3 |
| Eritrea | 3 |
| Ethiopia | 3 |
| Gambia | 3 |
| Guinea-Bissau | 3 |
| Nigeria | 3 |
| Sudan | 3 |
| Somalia | 3 |
| Chad | 3 |
| Angola | 2 |
| Central African Republic | 2 |
| Guinea | 2 |
| Kenya | 2 |
| Mali | 2 |
| Niger | 2 |
| Benin | 1 |
| Burkina Faso | 1 |
| Comoros | 1 |
| Egypt | 1 |
| Ghana | 1 |
| India | 1 |
| Iraq | 1 |
| Lesotho | 1 |
| Myanmar | 1 |
| Sierra Leone | 1 |
| Togo | 1 |
| Tanzania | 1 |

| ***Panel B: Aggregating 4 indicators of need^b^*** | |
| --- | --- |
| Cote d'Ivoire | 4 |
| D.R. Congo | 4 |
| Congo | 4 |
| Eritrea | 4 |
| Ethiopia | 4 |
| Gambia | 4 |
| Guinea-Bissau | 4 |
| Nigeria | 4 |
| Sudan | 4 |
| Somalia | 4 |
| Chad | 4 |
| Central African Republic | 3 |
| Angola | 2 |
| Comoros | 2 |
| Egypt | 2 |
| Guinea | 2 |
| India | 2 |
| Kenya | 2 |
| Lesotho | 2 |
| Mali | 2 |
| Niger | 2 |
| Benin | 1 |
| Burkina Faso | 1 |
| Ghana | 1 |
| Iraq | 1 |
| Myanmar | 1 |
| Papua New Guinea | 1 |
| Sierra Leone | 1 |
| Togo | 1 |
| Tanzania | 1 |
| Uzbekistan | 1 |
| Yemen | 1 |

^a^Maximum score = 3; ^b^Maximum score = 4.

## Robustness check using absolute indicators of need and funding

### Table S4. Country summary scores (maximum = 3) aggregating 3 indicators of need using absolute values of need and total disbursement amounts using 10% threshold

| **Country** | **Score** |
| --- | --- |
| Congo | 2 |
| Indonesia | 2 |
| Bhutan | 1 |
| Central African Republic | 1 |
| Cote d'Ivoire | 1 |
| Comoros | 1 |
| Djibouti | 1 |
| Gambia | 1 |
| Guinea-Bissau | 1 |
| Iraq | 1 |
| Sri Lanka | 1 |
| Myanmar | 1 |
| Niger | 1 |
| North Korea | 1 |
| Solomon Islands | 1 |
| Sierra Leone | 1 |
| Somalia | 1 |
| Sao Tome and Principe | 1 |
| Chad | 1 |
| Ukraine | 1 |
| Uzbekistan | 1 |
| South Africa | 1 |

### Table S5. Country summary scores (maximum = 3) aggregating 3 indicators of need using absolute values of need and total disbursement amounts using 25% threshold

| **Country** | **Score** |
| --- | --- |
| Congo | 3 |
| Laos | 3 |
| Somalia | 3 |
| Chad | 3 |
| Togo | 3 |
| Central African Republic | 2 |
| Cote d'Ivoire | 2 |
| Cameroon | 2 |
| Eritrea | 2 |
| Gambia | 2 |
| Indonesia | 2 |
| Myanmar | 2 |
| Niger | 2 |
| Sudan | 2 |
| Sierra Leone | 2 |
| South Sudan | 2 |
| Angola | 1 |
| Burundi | 1 |
| Burkina Faso | 1 |
| Bhutan | 1 |
| D.R. Congo | 1 |
| Comoros | 1 |
| Djibouti | 1 |
| Guinea-Bissau | 1 |
| Iraq | 1 |
| Sri Lanka | 1 |
| Lesotho | 1 |
| Mongolia | 1 |
| Mauritania | 1 |
| North Korea | 1 |
| State of Palestine | 1 |
| Solomon Islands | 1 |
| Sao Tome and Principe | 1 |
| Ukraine | 1 |
| Uzbekistan | 1 |
| Viet Nam | 1 |
| South Africa | 1 |

## Robustness check using reproductive health and health systems strengthening funding

### F**igure S3. Average reproductive health disbursements per capita vs. indicator of need, 2012-14**


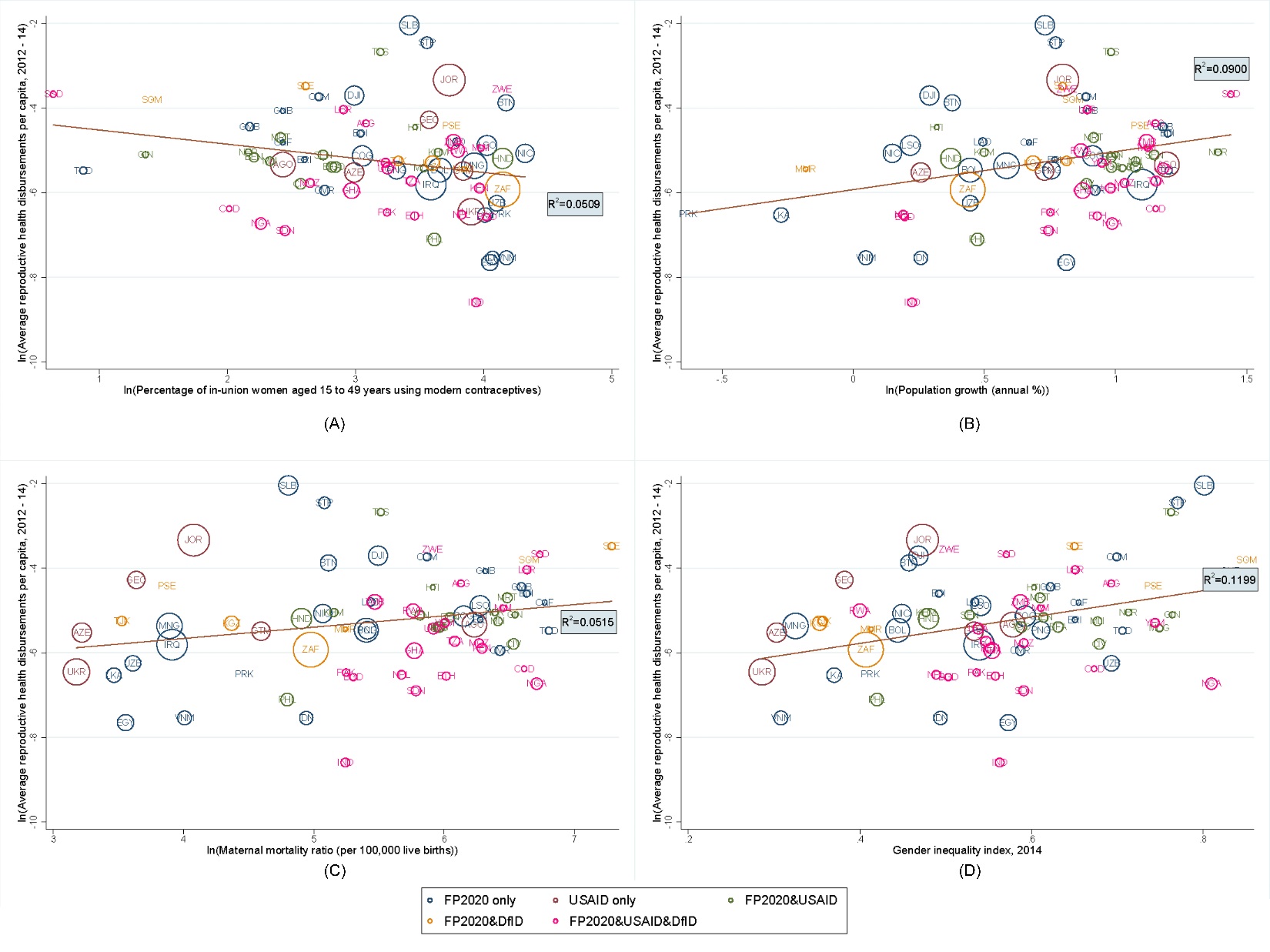


### Figure S4. Difference in rank of reproductive health disbursements per capita and rank of indicator of need


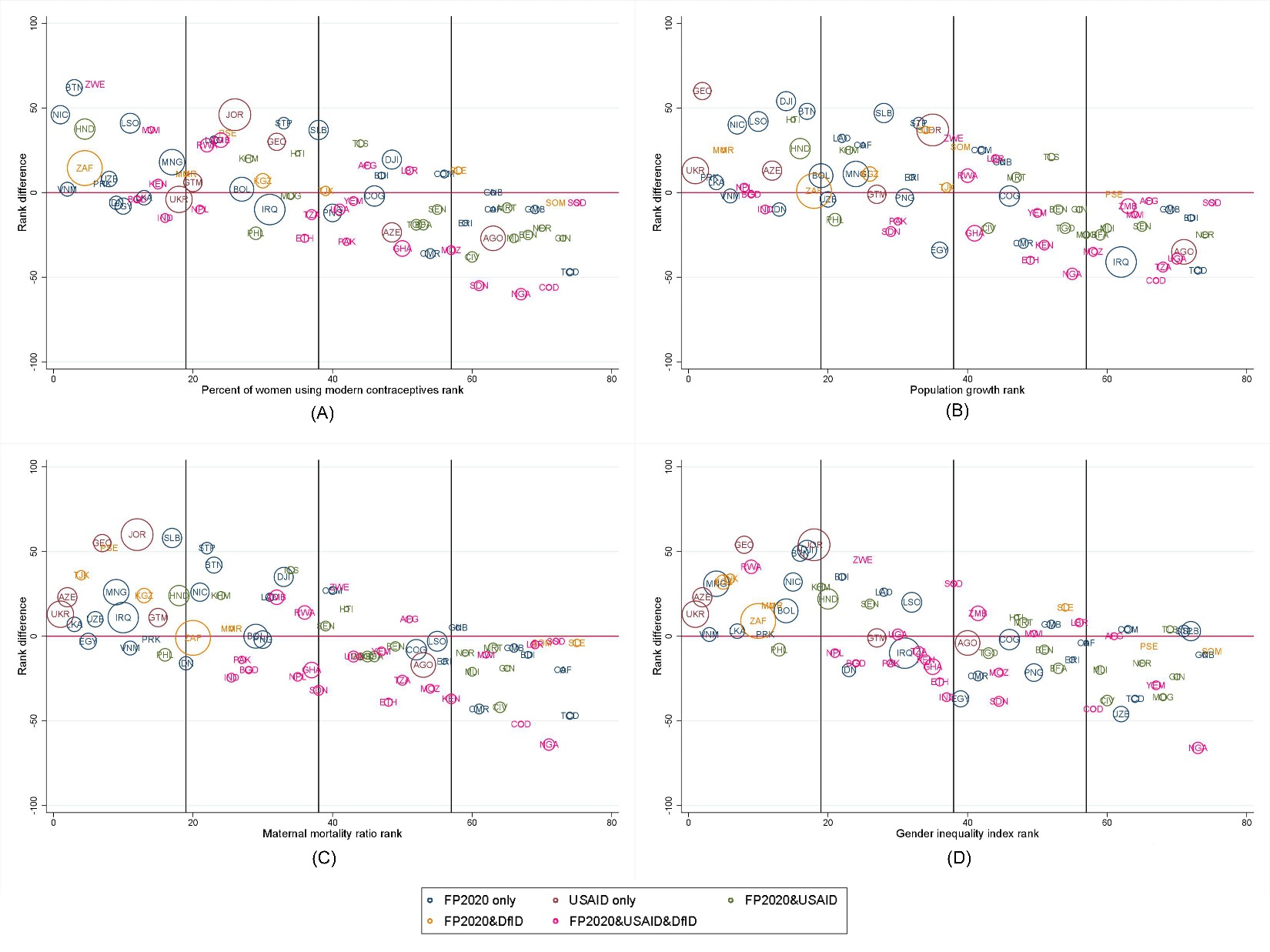


### Figure S5. Average reproductive health and health systems strengthening disbursements per capita vs. indicator of need, 2012-14


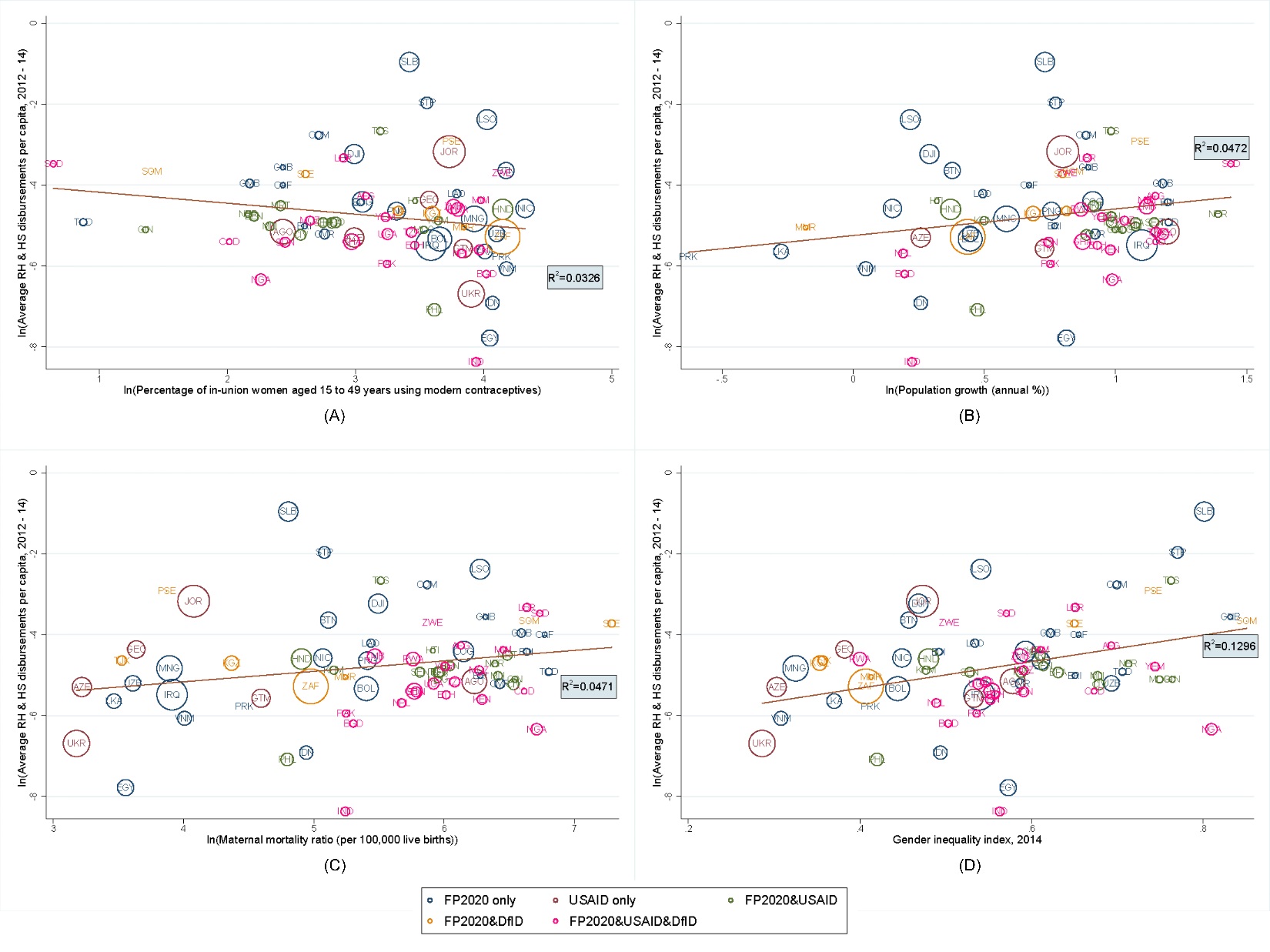


### Figure S6. Difference in rank of reproductive health and health systems strengthening disbursements per capita and rank of indicator of need


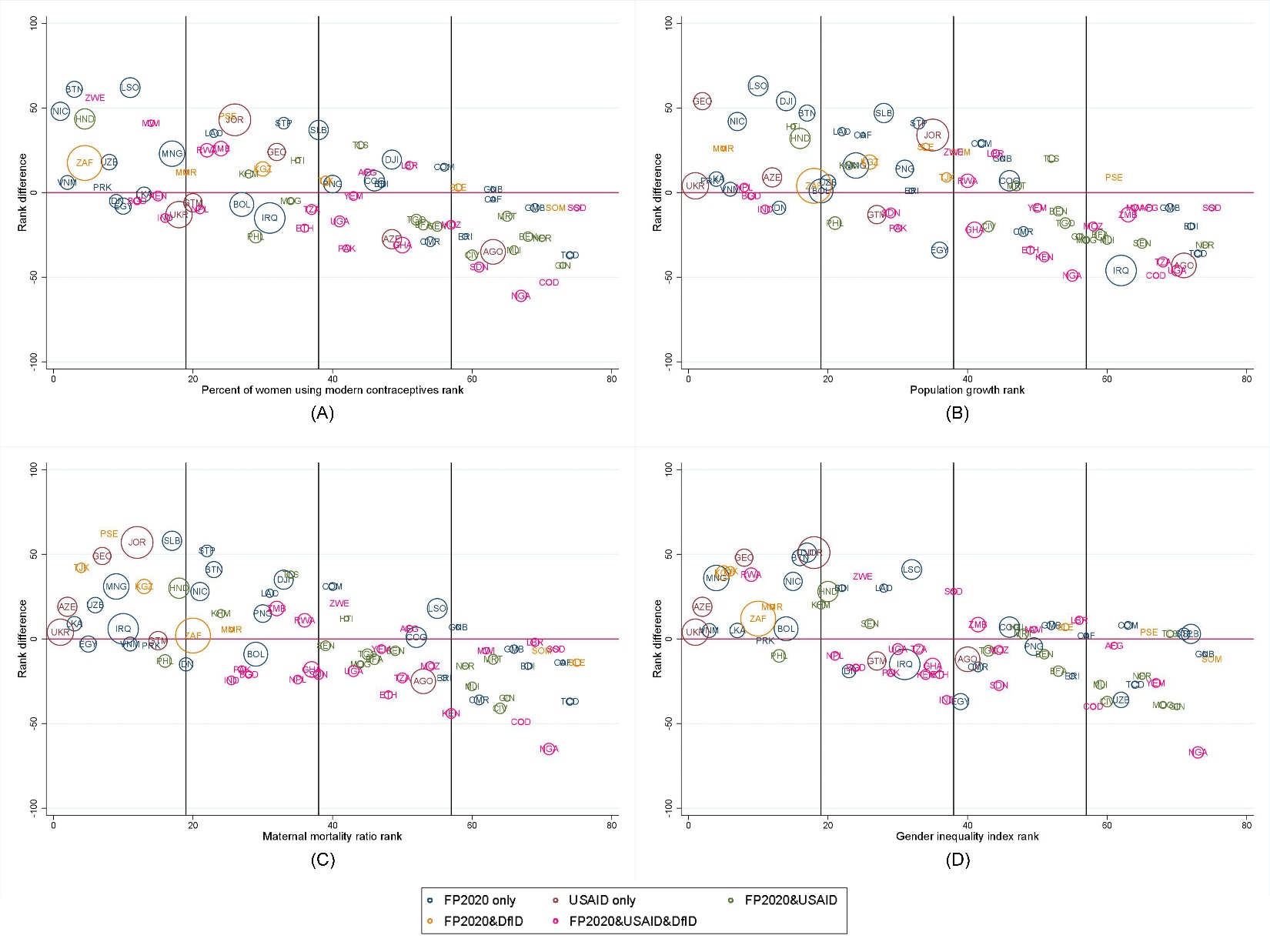


### Table S6. Country summary scores (maximum = 4) aggregating 4 indicators of need using reproductive health disbursements per capita with threshold of bottom 10%

| Country | Score |
| --- | --- |
| D.R. Congo | 4 |
| Nigeria | 4 |
| Chad | 4 |
| Cote d'Ivoire | 3 |
| Sudan | 3 |
| Cameroon | 2 |
| Ethiopia | 2 |
| Mozambique | 2 |
| Angola | 1 |
| Egypt | 1 |
| Ghana | 1 |
| India | 1 |
| Iraq | 1 |
| Kenya | 1 |
| Tanzania | 1 |
| Uganda | 1 |
| Uzbekistan | 1 |

### Table S7. Country summary scores (maximum = 4) aggregating 4 indicators of need using reproductive health and health systems strengthening disbursements per capita with threshold of bottom 10%

| Country | Score |
| --- | --- |
| D.R. Congo | 4 |
| Nigeria | 4 |
| Cote d'Ivoire | 3 |
| Guinea | 3 |
| Chad | 3 |
| Angola | 2 |
| Egypt | 2 |
| Kenya | 2 |
| Cameroon | 1 |
| Ethiopia | 1 |
| India | 1 |
| Iraq | 1 |
| Madagascar | 1 |
| Mali | 1 |
| Sudan | 1 |
| Tanzania | 1 |
| Uganda | 1 |
| Uzbekistan | 1 |
